# Cundurango by Its Friends

**Published:** 1872-04

**Authors:** 


### Cundurango by its Friends.

Through the kindness of Dr. John S. Perkins, of this city, we have been put in posession of some very interesting letters from persons whose names appear on Bliss, Keene & Co's certificates. The letters (five in number) do not seem to be very commendatory of the drug. Two of them from medical men deny ever giving any testimonials of its virtue whatever. Vice President Colfax says he always declines to sign certificates of any kind, but says that a *private* letter of his once got into print and was extensively published. The remaining two from persons outside of the profession do not give evidence of any remarkable cures. In fact both state that in the cases observed by them *no cure has been affected*, but they *think some* benefit has been derived from the use of Cundurango. The proprietors of Cundurango seem to have followed in the steps of all quack medicine venders, and secured certificates no matter by what means. One of the physicians alluded to, Dr. Fitch, of Chicago, says: "I have never authorized the use of my name in the connection you speak of, (Cundurango), and from this fact alone I am satisfied that the whole thing is a money making scheme and I may say a humbug."

